# Bruton’s Tyrosine Kinase Supports Gut Mucosal Immunity and Commensal Microbiome Recognition in Autoimmune Arthritis

**DOI:** 10.3389/fimmu.2022.748284

**Published:** 2022-03-29

**Authors:** Rachel H. Bonami, Christina E. Thurman, Sonam Verma, Camille S. Westlake, Lindsay E. Nyhoff, Bridgette B. Barron, Andrea Reboldi, Peggy L. Kendall

**Affiliations:** ^1^ Department of Medicine, Division of Rheumatology and Immunology, Vanderbilt University Medical Center, Nashville, TN, United States; ^2^ Department of Pathology, Microbiology and Immunology, Vanderbilt University Medical Center, Nashville, TN, United States; ^3^ Vanderbilt Institute for Infection, Immunology, and Inflammation (VI4), Nashville, TN, United States; ^4^ Department of Medicine, Division of Allergy, Pulmonary, and Critical Care, Vanderbilt University Medical Center, Nashville, TN, United States; ^5^ Department of Medicine, Division of Allergy and Immunology, Washington University School of Medicine, St. Louis, MO, United States; ^6^ Department of Pathology, University of Massachusetts Chan Medical School, Worcester, MA, United States

**Keywords:** B cells, IgA, Bruton’s tyrosine kinase, microbiome, autoimmunity

## Abstract

Bruton’s tyrosine kinase (*Btk*) deficiency preferentially eliminates autoreactive B cells while sparing normal humoral responses, but has not been studied in mucosal immunity. Commensal microbes and intact BTK signaling have been independently shown to be essential for arthritis development in K/BxN mice. Here, we examine how BTK-mediated signaling interfaces with the gut microbiome. *Btk*-deficient K/BxN mice were found to have small Peyer’s Patches with reduced germinal center and IgA class-switched B cells. IgA-switched plasma cells in small intestines were reduced, especially in villi of *Btk*-deficient mice. IgH CDR3 sequencing showed similar V gene diversity and somatic hypermutation frequency despite *Btk* deficiency but showed reduced CDR3 amino acid polarity, suggesting potential qualitative differences in the gut plasma cell repertoire. Small intestinal IgA was low and IgA coating of commensal bacteria was reduced. IgA-seq showed a shift in small intestinal microbes that are normally IgA-coated into the uncoated fraction in *Btk*-deficient mice. Overall, this study shows that BTK supports normal intestinal IgA development in response to commensals.

This manuscript was previously published as a preprint at: https://www.biorxiv.org/content/10.1101/2021.03.10.434762v2.

## Introduction

More IgA is produced daily than all other antibody isotypes combined, highlighting its importance to host health (1). IgA-deficient humans are relatively resistant to infection, presumably due to functional compensation by secreted IgM, but they do have a propensity for autoimmunity (2–4). B cell signaling contributions to IgA development require further study. B cells in mucosal lymphoid tissues undergo IgA class-switch and affinity maturation, giving rise to plasma cells that migrate to the small intestinal lamina propria. B2 B cells in Peyer’s patches undergo T-dependent responses in germinal centers (GCs) while B1 cells that traffic from the peritoneal cavity are more likely driven by T-independent responses without requiring GCs (5–7). It is thought that commensal recognition by the immune system *via* IgA occurs primarily through T-independent responses, whereas pathogens more typically elicit T-dependent responses (6). This is a reciprocal process as microbes are required for adaptive immune structure formation in the gut; germ-free mice fail to develop normal Peyer’s patch structures or IgA-secreting plasma cells (8). IgA coating of microorganisms can block their attachment to host epithelia, thereby helping to maintain barrier function, or alternatively promote their retention in the mucous layer (9, 10).

B cell functions depend upon signaling through the B cell receptor (BCR). The B cell signaling protein Bruton’s tyrosine kinase (BTK) serves as a “rheostat” in mature B cells, acting to propagate and amplify signals. Anti-citrullinated protein antibody (ACPA)-positive, but not ACPA-negative rheumatoid arthritis patients show elevated BTK phosphorylation relative to healthy controls (11), highlighting a link between overzealous BTK signaling and ACPA production. Murine *Btk*-deficiency reduces GC formation and T-independent immune responses, as well as autoantibodies, but T-dependent responses can be elicited, and IgG levels are near normal (12–18). In humans, BTK is necessary for B cells to mature beyond the bone marrow, making study of *Btk*-deficiency in mature peripheral B cells difficult (19).

*Btk*-deficiency protects against autoimmune arthritis in K/BxN mice (12). This is consistent with a role for *Btk* in autoreactive B lymphocyte development and function, as autoantibodies provoke arthritis in this model (20). Commensal microorganisms are also central to autoimmune arthritis development, as germ-free or antibiotic-treated K/BxN mice are protected from spontaneous disease development (21). Conversely, segmented filamentous bacteria (SFB) (also known as *Candidata savagella*) mono-colonization drives autoimmune arthritis, confirming commensal bacteria can trigger disease (21).

Commensal bacteria exposure shapes the gut B cell repertoire (22). The impact of altered B cell receptor signaling on B lymphocyte responses is incompletely understood. Clinical applications for BTK inhibitors continue to increase and include autoimmune diseases such as rheumatoid arthritis (23). Serum IgA was found to be normal in *Btk*-deficient mice (14). Similarly, IgA^+^ B220^-^ cells in the large intestine (colon) were not changed by loss of BTK in a DSS-induced colitis model (24). B cell changes in the small intestine were not investigated in this model (24). Peyer’s patches in the small intestine are a major site of gut B cell activation which support the development of gut IgA^+^ plasma cells. The requirement for BTK in preserving normal small intestinal populations of B lymphocytes has been largely unexplored. We therefore investigated how *Btk*-deficiency impacts B lymphocyte populations and responses to commensal bacteria in the gut in the setting of autoimmune arthritis, with particular focus on small intestinal B cells and plasma cells. Peyer’s patch GCs are dramatically reduced. B1a cells are absent as expected. IgA^+^ plasma cells are present in *Btk*-deficient K/BxN mice but are significantly reduced in the villi where they normally predominate. IgA^+^ plasma cells do not show diminished BCR diversity or somatic hypermutation, based on CDR3 analysis, although amino acid polarity is altered. However, IgA is reduced and IgA-coated bacterial composition is shifted in the small intestine of *Btk*-deficient mice. These data show impaired B cell receptor signaling shifts the dynamic interplay between host B lymphocytes and commensal bacteria in the gut.

## Materials and Methods

### Animals and Disease Assessment

*Btk^-/-^*/NOD mice were backcrossed to the NOD strain >20 generations and were generated as described previously (13). KRN C57BL/6 mice were a gift from Cristophe Benoist and Diane Mathis (Harvard Medical School, Boston MA). KRN male mice were bred to *Btk^+/-^*/NOD female mice to generate *Btk^+/y^*/K/BxN and *Btk^-/y^*/K/BxN littermates. Mice were scored for arthritis beginning at weaning by the Chondrex mouse arthritis scoring system (https://www.chondrex.com/documents/Mouse%20CIA.pdf). Paw thickness of the hind footpad was measured using a dial gauge (13-159-9, Swiss Precision Instruments). Male mice were used in all studies. All mice were housed under specific pathogen-free conditions, or in the case of *P. distasonis* infection, in the ABSL-2 facility, and all studies were approved by the institutional animal care and use committee of Vanderbilt University, fully accredited by the AAALAC.

### Mammalian Cell Isolation, Flow Cytometry, and Antibodies

The entire small intestine (duodenum, jejunum, and ileum) was dissected and intestinal contents were flushed by lavage with 5mL 1X PBS + EDTA-free Complete Mini Protease Inhibitor Cocktail Tabs (Roche). Peyer’s patches were dissected from the small intestine and macerated in HBSS + 10% FBS. Lamina propria cells were isolated after Peyer’s patches were removed from the entire small intestine as follows. Intestines were cut longitudinally and mucus was gently scraped off and discarded. Tissue was cut into ~0.5 cm pieces and incubated with HBSS + 5% bovine calf serum (BCS) + 2 mM EDTA at 37°C for 45 min while shaking. Recovered tissue was washed, minced, and incubated with 1.5 mg/ml collagenase VIII (from *Clostridium histolyticum*, Sigma-Aldrich) and 100 U DNase I (Sigma-Aldrich) at 37°C for 25 min while shaking. Cells were vortexed for 20 s and HBSS + 5% BCS was added, cells were pelleted, and resuspended in 40% Percoll (GE Healthcare). 70% Percoll was underlayed and the interface layer was recovered after centrifugation at 300 x g for 20 min at 4°C. Cells were pelleted, resuspended, and counted.

Cells were stained for flow cytometry in 1X PBS containing 0.1% sodium azide, 0.02% EDTA, and 0.05% BCS using the following reagents and reactive antibodies: B220 (6B2), CD19 (1D3), CD95/FAS (JO2), CD138 (281-2), GL7, Igκ (187.1), or Ghost Dye (BD Biosciences, eBioscience, or Tonbo Biosciences), and/or IgA (1040-09, 1040-02, or 1040-31, Southern Biotech). Lamina propria cells were stained intracellularly for CD138, as this protein was cleaved from the cell surface during the isolation protocol (not shown). In addition to surface Igκ and IgA staining, lamina propria cells were also stained intracellularly with Igκ and IgA-reactive antibodies to aid identification of surface BCR-negative plasma cells. The Cytofix/Cytoperm kit was used for intracellular permeabilization and staining (BD Biosciences). Samples were acquired using a BD Biosciences LSR II flow cytometer and FlowJo software (Tree Star, Inc) was used for data analysis.

### Fecal and Small Intestinal Lavage Sample Preparation and ELISA

Freshly collected feces were initially weighed and resuspended at 0.1mg/mL in 1X PBS containing EDTA-free Complete Mini Protease Inhibitor Cocktail Tabs (Roche) per the manufacturer’s instructions. Samples were subsequently spun at 4000 RPM for 10 min., supernatant was removed and spun for an additional 10min and resulting supernatant was sterile filtered through 0.22μm filters and stored at -20°C.

To collect small intestinal lavage for ELISA, the entire small intestine (duodenum, jejunum, and ileum) was collected from each mouse and immediately flushed with 5 mL 1X PBS containing EDTA-free Complete Mini Protease Inhibitor Cocktail Tabs (Roche) per the manufacturer’s instructions to normalize sample collection across mice. Samples were subsequently spun at 400 x g for 5 min. and then passed through a 70 µm nylon cell strainer to remove large debris. Supernatant was then spun at 8000 x g for 10 min. to pellet bacteria and resulting supernatant was sterile filtered through 0.22μm filters and stored at -20°C prior to ELISA.

For detection of total IgA, plates were coated with goat anti-mouse IgM + IgG + IgA (H+L) (SouthernBiotech) diluted in 1X PBS, followed by blocking with 10% non-fat dry milk. Sera were diluted 1:50,000 in 1X PBS + 0.05% tween and incubated 2h at room temperature. Bound IgA or IgM antibody was detected by incubation with goat anti-mouse IgA-alkaline phosphatase (α chain-specific, SouthernBiotech) or goat anti-mouse IgM-alkaline phosphatase (μ chain-, SouthernBiotech), respectively diluted in veronal buffered saline (142 mM NaCL + 5 mM sodium barbital) + 15mM sodium azide + 0.5% FBS. Plates were washed 4X with 1X PBS + 0.05% tween after each incubation step. 10mg/mL *p*-nitrophenyl phosphate substrate (Sigma-Aldrich) was added and O.D. was read at 405 nm *using a* Microplate Autoreader (Bio-Tek Instruments). Intestinal IgA was quantified based on a standard curve that was generated using Purified Mouse IgA, κ Isotype Control (BD Biosciences).

### Small Intestine Immunostaining

Small intestines were isolated from 8-11 wk-old *Btk*-sufficient and *Btk*-deficient K/BxN males, cleaned using chilled 1X PBS, and dissected longitudinally. Small intestines were divided into four parts and enumerated from proximal (duodenal) to distal (ileal) as SI1, SI2, SI3, and SI4. A swiss-roll was made from each section, fixed in 4% paraformaldehyde followed by a series of dehydration in 5-20% sucrose., and frozen in O.C.T. Compound (Tissue-Tek). 7μm sections were cut from the duodenum (SI1) and ileum (SI4) and used for immunostaining. Frozen sections were fixed using chilled 50:50 mixture of methanol and acetone for 10 min at room temperature, washed with distilled water followed by 1X PBS, then blocked using 1% bovine serum albumin for 1 h. Sections were then stained overnight at 4°C using primary antibodies for epithelial cell adhesion molecule (EpCAM) (1:400) Thermo Fisher Scientific, #21050-1-AP) and anti-IgA (1:200) (Southern Biotech, #1165-01). After washing with 1X PBS three times, anti-rabbit Alexa 488 (1:800) and anti-rat Alexa 633 (1:400), (Thermo Fisher Scientific, #A-11034 and #A-21094, respectively) were added for 1h at room temperature, followed by washing with 1X PBS. Counterstaining was performed using Hoechst dye and fixation in ProLong Diamond Antifade mounting medium. Images were captured from non-overlapping areas using a Zeiss LSM880 Confocal Laser Scanning Microscope with Airyscan. Overall, 136 images were scored in parallel by two blinded reviewers, with any differences of opinion resolved by a third blinded reviewer. IgA^+^ cells were enumerated for villi and lamina propria separately, from 0 to 5 based on frequency of IgA^+^ cells among DAPI^+^ cells using a blind evaluation strategy (0 = 0% IgA; 1 = 1-20% IgA; 2 = 20-40% IgA; 3 = 40-60% IgA; 4 = 60-80% IgA; 5 = 80-100% IgA). Scores for villi and lamina propria were added for each image to yield total IgA for that image.

### Plasma Cell IgH Sequencing

Lamina propria cells were prepared from freshly isolated small intestines as outlined above. Cells were stained for surface proteins, fixed with 0.5% paraformaldehyde for 5 minutes (to retain surface antibodies but limit potential DNA cross-linking), washed, and permeabilized with 0.1% saponin in 1X PBS containing 10% BCS, followed by staining for intracellular proteins using antibody clones outlined above. A BD FACSAria III was used to sort IgA^+^ plasma cells that were resuspended in 1X PBS (live B220^-^ CD19^-^ intracellular IgA^+^ surface IgA^-^ CD138^+^ cells). Cell pellets were stored at -80°C prior to DNA extraction. DNA was isolated as a single batch using the QIAamp DNA Micro Kit (Qiagen) per the manufacturer’s instructions. Mouse IgH CDR3 PCR amplification and sequencing was performed using the ImmunoSeq assay and sequence data were demultiplexed/filtered/processed by Adaptive Biotechnologies (Seattle, WA) (25). This bulk sequencing approach uses a synthetic immune repertoire as an in-line template control and removes singlet sequences during data processing to enable normalization for both PCR efficiency and amplification bias to ensure processed data correspond to the absolute number of input molecules (26). IMGT/HighV-QUEST (https://www.imgt.org/HighV-QUEST/) was used to assign gene identities and identify mutations among sequences with productive rearrangements. VDJtools (https://vdjtools-doc.readthedocs.io/en/master/index.html) was used to compare repertoire diversity and CDR3 properties between the two groups.

### Microbiome Sample Preparation and Sequence Analysis

Small intestines were dissected and intestinal contents were flushed by lavage as above and centrifuged at 400 x g for 5 min. Supernatant was then centrifuged at 8000 x g for 10 min to pellet bacteria. The bacterial pellet was resuspended in 1X PBS + 0.25% BSA + 5% normal goat serum (NGS), normalized for roughly similar numbers of bacteria based on O.D. 600 nm readings, and stained with Fc Block (BD Biosciences, clone 2.4G2), followed by SYTO BC (ThermoFisher) and goat anti-mouse IgA. Cells were sorted on a BD FACSAria III and cells were pelleted and stored at -80°C. DNA extraction, microbiome sequencing, and analysis was performed as a single batch by Microbiome Insights. Bacterial 16S rRNA v4 regions were PCR-amplified as in (27). Amplicons were sequenced with an Illumina MiSeq using the 250-bp paired-end kit (v.2). Sequences were denoised, taxonomically classified (Greengenes v. 13_8 reference database), and clustered into 97%-similarity operational taxonomic units (OTUs) using Mothur, v. 1.39.5 (28). Potential contamination was addressed by co-sequencing DNA amplified from specimens and from n=4 template-free controls and n=4 extraction kit reagents processed the same way as the specimens. Two positive controls, consisting of cloned SUP05 DNA, were also included (number of copies = 2*10^6). OTUs were considered putative contaminants (and were removed) if their mean abundance in controls reached or exceeded 25% of their mean abundance in specimens.

### Statistical Analysis

GraphPad (Prism) was used for statistical comparisons of flow cytometry, ELISA, and disease study data. Immune repertoire and microbiome statistical analysis approaches are detailed in the relevant figure legends.

## Results

### IgA-Switched and GC B Cells Are Reduced in Peyer’s Patches of *Btk*-Deficient K/BxN Mice

**Figure 1:**
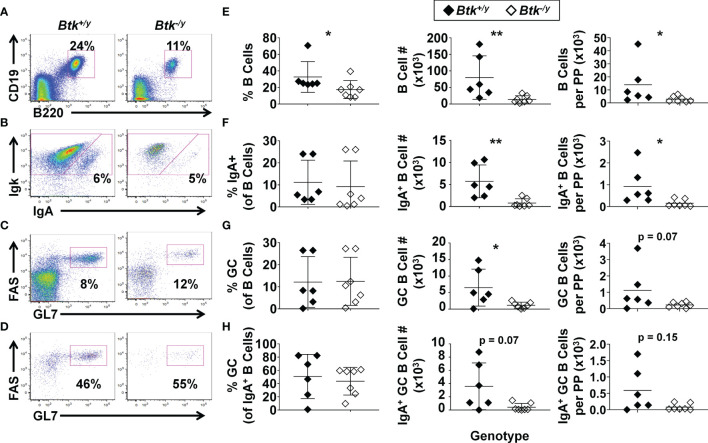
*Btk* absence leads to reduced numbers of GC and IgA class-switched B cells in the Peyer’s patches in K/BxN mice. Peyer’s patches were harvested from 8-9 wk-old K/BxN (black) and *Btk* ^-/y^/K/BxN (white) littermates and cells isolated from all Peyer’s patches dissected from each mouse were counted; n ≥ 6 mice per group, n = 5 independent experiments, as in Methods. Flow cytometry was used to identify the indicated subsets. **(A-D)** Representative plots for K/BxN (left) and *Btk* ^-/y^/K/BxN (right) mice depict **(A)** B cells (B220^+^ CD19^+^) among live singlet lymphocytes, **(B)** IgA^+^ B cells among B220^+^ CD19^+^ live singlet lymphocytes, **(C)** GC B cells (GL7^high^ FAS^high^) among B220^+^ CD19^+^ live singlet lymphocytes, and **(D)** GC B cells among IgA^+^ B220^+^ CD19^+^ live lymphocytes. **(E-H)** Frequencies (left), numbers across all Peyer’s patches harvested per mouse (middle), and numbers divided by the number of Peyer’s patches harvested per mouse (right) are plotted for individual mice to show **(E)** total B cells, **(F)** IgA^+^ B cells, **(G)** GC B cells, and **(H)** IgA^+^ GC B cells. *p < 0.05, **p < 0.01, Mann-Whitney U test.

*Btk*-deficiency protects against several spontaneous autoimmune diseases in mouse models (12, 13) and clinical trials investigating the efficacy of BTK inhibitors in human autoimmune disease are ongoing (reviewed in (29)). The small intestine is a major site of B cell activation in the gut, yet the impact of *Btk* deficiency on B lymphocyte development and function at this site has not been investigated. We previously reported that *Btk*-deficiency protects against autoimmune arthritis in K/BxN mice, a well-studied model of rheumatoid arthritis in which gut commensal bacteria are essential disease triggers (12, 21). We therefore investigated how *Btk* loss alters B cell numbers, isotype switch, and differentiation in the Peyer’s patches and lamina propria of the small intestine in the setting of autoimmune arthritis. Total numbers of B cells, IgA^+^ B cells, and GC B cells were significantly reduced in Peyer’s patches of *Btk*-deficient K/BxN mice compared to *Btk*-sufficient K/BxN littermate controls (**Figure 1**). Significant reductions in these populations were still seen when numbers were normalized to the number of Peyer’s patches collected, with the exception of GC B cells, which still trended lower with BTK loss (p = 0.07). In contrast to the observed numeric differences, the frequency of Peyer’s patch B cells that differentiated into GC B cells or class-switched to IgA was not significantly different between *Btk^+/y^* and *Btk^-/y^* K/BxN mice; the average frequency of total B cells or IgA^+^ B cells that acquired the GC phenotype was ~10% and 40%, respectively, regardless of BTK expression (**Figure 1**). Taken together, these data suggest *Btk*-deficiency reduces Peyer’s patch B cell numbers overall, but does not block B cell class switching to IgA or participation in GC reactions for Peyer’s patch B cells.

### B1a B Cells Are Reduced in *Btk*-Deficient K/BxN Mice

**Figure 2:**
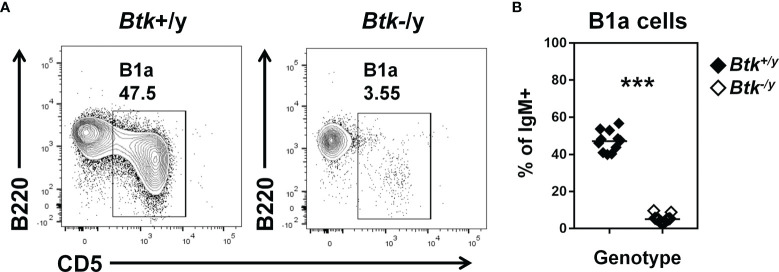
B1a B cells are reduced in *Btk*-deficient K/BxN mice. Flow cytometry identifies B1a B cells in peritoneal lavage of K/BxN (black) and *Btk^-/y^*/K/BxN (white) littermates, n ≥ 11 mice per group, n = 3 independent experiments. **(A)** Representative plots are gated on IgM^+^ live singlet lymphocytes and indicate the frequency of B1a B cells (B220^low^ CD5^+^). **(B)** B1a B cell frequency is plotted for individual mice, ***p < 0.001, Mann-Whitney U test.

IgA-secreting plasma cells in the small intestine derive from B2 cells in the Peyer’s patches and lamina propria and B1 cells in the peritoneal cavity (5, 6, 30). B1a cells are dramatically reduced in *Btk*-deficient mice, while B1b cell numbers are normal (14). Consistent with observations in a non-autoimmune strain (14), B1a B cells were strongly reduced in the peritoneal cavity of *Btk*-deficient K/BxN mice (**Figure 2**). Thus, both peritoneal B1a and Peyer’s patch B2 B cell plasma cell precursors are reduced in *Btk*-deficient K/BxN mice.

### IgA^+^ Plasma Cells Persist in *Btk*-Deficient K/BxN Mice but Are Reduced in Small Intestinal Villi

**Figure 3:**
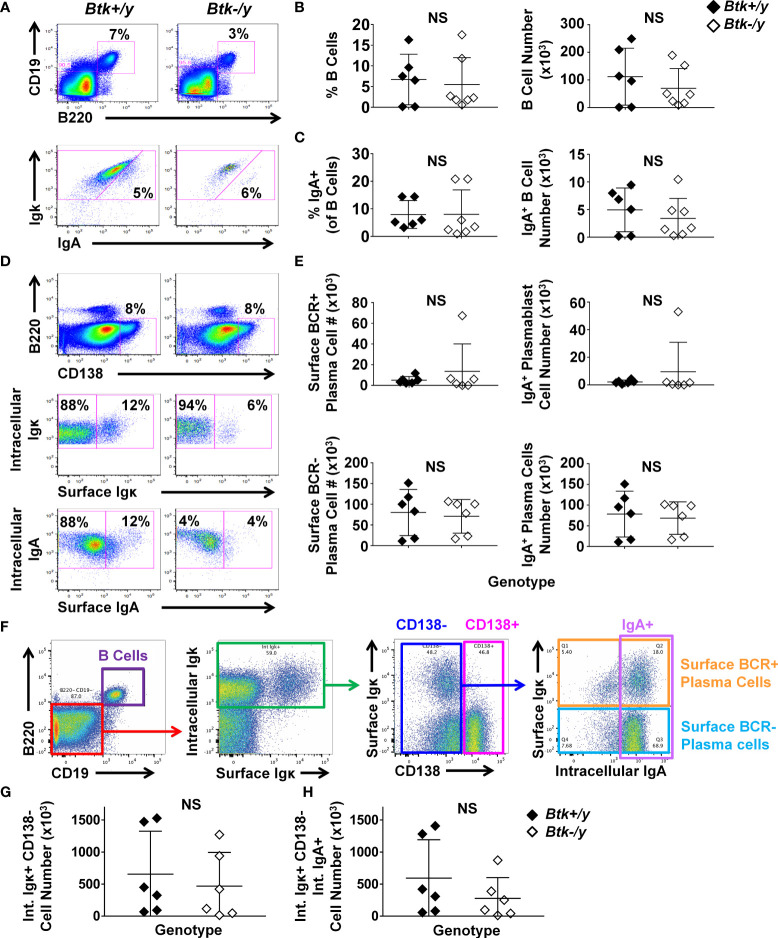
IgA^+^ plasma cells persist in the lamina propria of *Btk*-deficient K/BxN mice. Lamina propria cells were harvested from the entire small intestine of 8-9 wk-old K/BxN (black) and *Btk^-/y^*/K/BxN (white) littermates as in Methods and cells were counted; n ≥ 6 mice per group, n = 5 independent experiments. Flow cytometry was used to identify the indicated subsets. **(A)** Representative plots depict total B cells (B220^+^ CD19^+^) among live singlet lymphocyte gated cells in K/BxN (left) and *Btk^-/y^*/K/BxN (right) mice (top) and IgA^+^ B cells among total B cells (bottom). **(B)** Total B cell frequencies (left) and numbers (right) and **(C)** IgA^+^ B cell frequencies (left) and numbers (right) are shown. **(D, E)** Plasma cells were identified among B220^-^ CD19^-^ intracellular Igκ^+^ CD138^+^ live singlet cells as surface BCR-positive (surface Igκ^+^), surface BCR-negative (surface Igκ^-^), surface BCR-positive IgA (surface Igκ^+^, surface IgA^+^), and surface BCR-negative IgA (surface Igκ^-^, intracellular IgA^+^). **(D)** Representative plots depict the indicated subsets in K/BxN (left) and *Btk^-/y^*/K/BxN (right). **(E)** Cell subset numbers are shown for surface BCR^+^ plasma cells (top left), surface BCR^+^ IgA^+^ plasma cells (top right), surface BCR^-^ plasma cells (bottom left), and surface BCR^-^ IgA^+^ plasma cells (bottom right). **(F–H)** CD138- cells with a plasma cell phenotype were further examined as follows. **(F)** Representative gating scheme shows B220^+^ CD19^+^ and B220^-^ CD19^-^ populations among live singlet lymphocytes (left). B220^-^ CD19^-^ cells (red) were further gated on intracellular Igκ^+^ cells (green, middle, left), followed by identification of CD138^-^ (blue) and CD138^+^ (pink) cells (middle, right). Intracellular IgA expression was examined among CD138^-^ cells (right, lavender), among which surface Igκ expression was used to delineate surface BCR-positive (orange) and surface BCR-negative (light blue) plasma cells. **(G, H)** Numbers of live singlet lymphocytes that were B220^-^ CD19^-^ intracellular Igκ^+^ CD138^-^ cells are shown for **(G)** total cells or **(H)** IgA-switched cells. No significant differences were observed between K/BxN and *Btk^-/y^*/K/BxN groups, Mann-Whitney U test. NS, Not significant.

IgA^+^ GC B cells in Peyer’s patches and B1a cells, which serve as intestinal plasma cell precursors, were strikingly reduced (**Figures 1**, **2**). Lamina propria B cells can also serve as IgA plasma cell precursors (30). We therefore tested whether *Btk* loss also reduced IgA-switched B cells and plasma cells in the lamina propria using flow cytometry of cells extracted from the entire small intestinal luminal layer after removal of Peyer’s patches. The number of total B cells and IgA-switched B cells present in the lamina propria was not altered by *Btk*-deficiency (**Figures 3A–C**). Downregulation of the classic B cell markers, B220 and CD19 complicates identification of plasma cells. Furthermore, CD138 expression was previously shown to fluctuate with disease on plasma cells in a mouse model of multiple sclerosis (31). We therefore used intracellular Igκ positivity to help identify potential antibody-secreting cells, as 95% of mouse B cells express Igκ (32). We observed two populations of CD19^-^ B220^-^ CD138^+^ cells which were either surface BCR-positive or surface BCR-negative, which were analyzed separately. Prior studies showed this surface BCR-positive subset to lack cell proliferation markers such as Ki67, suggesting they were plasma cells, not plasmablasts (33). We will therefore designate both subsets as plasma cells but acknowledge the BCR-positive subsets may also contain plasmablasts, as our present study does not distinguish whether they are blasting. The majority of B220^-^ CD19^-^ CD138^+^ plasma cell subsets in the lamina propria were IgA class-switched in both *Btk*-sufficient and deficient K/BxN mice (**Figures 3D, E**). CD138^+^ IgA^+^ plasma cell numbers were extremely low and not reduced by *Btk* loss. Among CD19^-^ B220^-^ intracellular Igκ^+^ cells, we also observed a CD138^-^ population, a subset of which expressed intracellular IgA (**Figure 3F**). This population may be an additional population of antibody-secreting cells in the lamina propria and was therefore analyzed separately. The numbers of CD19^-^ B220^-^ intracellular Igκ^+^ CD138^-^ cells trended lower but was not significantly reduced in *Btk*-deficient relative to *Btk*-sufficient K/BxN mice, both for total numbers (**Figure 3G**, p = 0.48) and in the IgA-switched population (**Figure 3H**, p = 0.31). Thus, IgA-switched antibody-secreting cells are present in the lamina propria despite marked reductions in Peyer’s patch GC and B1a B cells in *Btk*-deficient mice. Preservation of lamina propria B cells, which presumably reside in isolated lymphoid follicles, could help explain the preservation of IgA-switched antibody-secreting cells observed in *Btk*-deficient mice.

**Figure 4:**
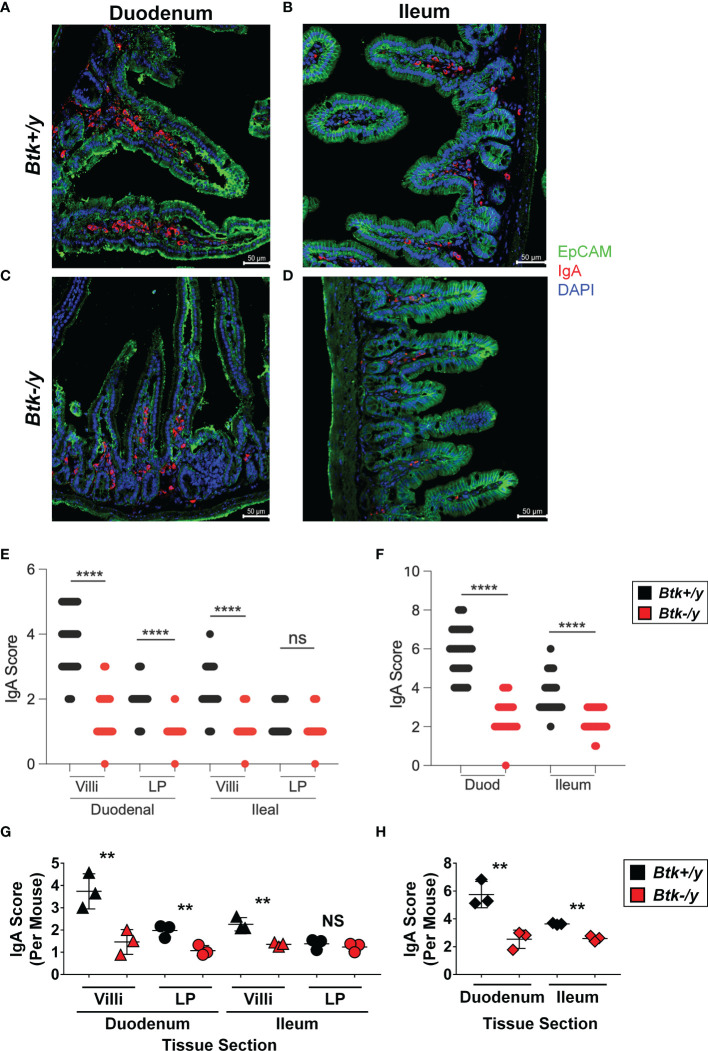
*Btk*-deficiency alters IgA plasma cell localization in the small intestine of K/BxN mice. Small intestine was collected from 8-11wk-old *Btk^+/y^* (black) and *Btk^-/y^* (red) K/BxN mice (n = 3-4 mice per group) as described in Materials and Methods. Immunostaining for IgA (red), EpCAM (green), and DAPI (blue) was performed to identify IgA^+^ cells in the small intestine villi and lamina propria. Representative images of non-overlapping areas of sections from each mouse are shown for **(A)** *Btk^+/y^*/K/BxN and **(B)** *Btk^-/y^*/K/BxN mouse S1 duodenal sections (*Btk^+/y^* S1 mean/min/max = 13/7/24 images per mouse; *Btk^-/y^* S1 mean/min/max = 9/6/12 images per mouse). Representative images for **(C)** *Btk^+/y^*/K/BxN and **(D)** *Btk^-/y^*/K/BxN mouse S4 ileal sections. (*Btk^+/y^* S4 mean/min/max = 17/10/23 images per mouse; *Btk^-/y^* S4 mean/min/max = 9/8/12 images per mouse). Scale bar represents 50 µm. **(E, F)** Analysis of IgA^+^ plasma cell frequency in **(E)** duodenal and ileal villi and lamina propria (LP) and **(F)** duodenal and ileal overall tissues as measured by IgA score on immunofluorescent sections. Each dot represents a single image and data are compiled from n = 3-4 mice per genotype. ****p < 0.0001 ANOVA with Bonferroni correction. **(G, H)** Average IgA scores for each mouse are plotted for **(G)** villi and lamina propria (LP) or **(H)** total duodenal (S1) or ileal (S4) sections. **p < 0.01, NS, not significant, two-tailed t test with Welch’s correction.

Flow cytometry of total small intestinal lamina propria using this method has several limitations, including unrecognized inclusion of intraepithelial lymphocytes, small lymphoid follicles, and blood vessels. Furthermore, the majority of lamina propria cells that had an apparent plasma cell phenotype (CD19^-^ B220^-^ intracellular Igκ^+^ intracellular IgA^+^) stained negatively for the canonical plasma cell marker, CD138 (**Figure 3E** vs. **Figure 3H**). We therefore used immunofluorescent staining to better identify and locate IgA^+^ plasma cells *in situ* (**Figure 4**). EpCAM (green) stains the epithelia, DAPI (blue) marks cell nuclei, and IgA (red) identifies plasma cells as typical large cells in villi and lamina propria ((34), **Figures 4A–D**). Plasma cells are normally found in greatest abundance in the duodenum, and gradually reduce distally, with the fewest found in the ileum. We therefore chose duodenal and ileal sections as representative for analysis. Sections were scored for proportion of IgA^+^ cells among all DAPI^+^ cells in villi or lamina propria areas of each section. IgA^+^ plasma cells were found to be abundant in *Btk*-sufficient K/BxN duodenal sections and were particularly enriched in the intestinal villi, where they are well-positioned for IgA transepithelial transport (**Figures 4E, G** **)**. Villi in the ileum of *Btk*-sufficient K/BxN similarly contained more IgA^+^ cells than the lamina propria (**Figures 4E, G** **)**, although fewer IgA^+^ cells were observed in the ileum overall than in the duodenum (**Figures 4F, H** **)**. In contrast, the overall proportion of IgA^+^ cells in *Btk*-deficient sections were significantly lower than in *Btk*-sufficient mice, which was most pronounced in duodenal villi (**Figures 4E–H**). The only area in which there was no difference in IgA^+^ cells was the lamina propria of the ileal sections, where the lowest numbers of IgA^+^ cells were also found in *Btk^+/y^* controls (**Figure 4G**). In addition, the overall condition of the intestinal epithelium and villi appeared subjectively less robust, with the epithelial layer staining more poorly, and villi often appearing shorter, although these features were not quantified in this study. Thus, IgA^+^ plasma cells in *Btk*-deficient K/BxN mice are not appropriately localized to small intestinal villi.

### *Btk* Loss Reduces Small Intestinal but Not Serum Levels of IgA in K/BxN Mice

**Figure 5:**
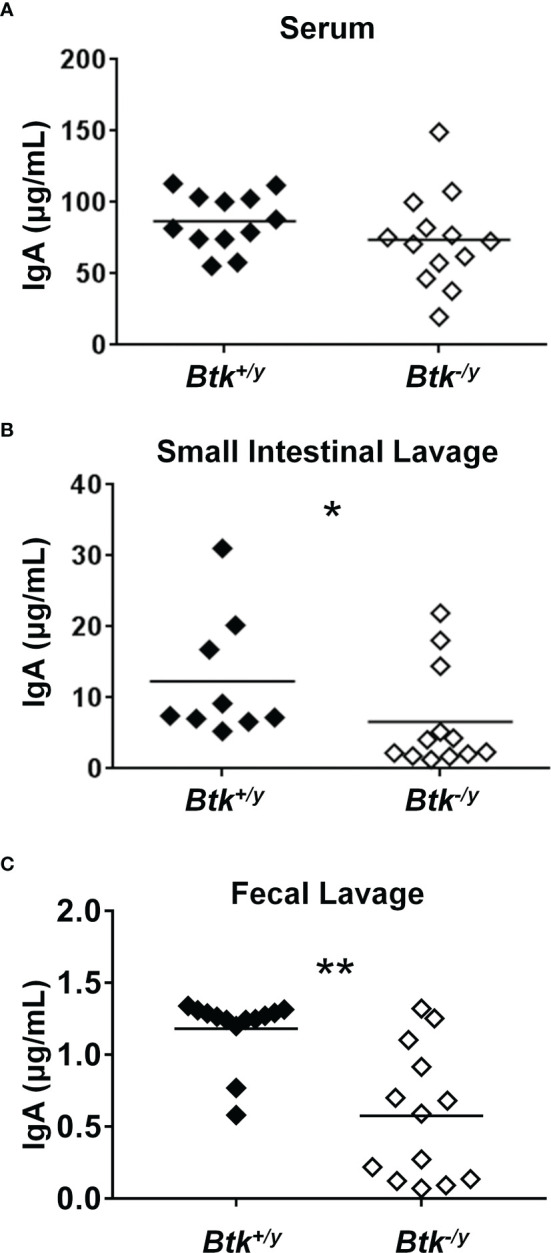
IgA is reduced in the small intestine and feces, but not serum of *Btk*-deficient K/BxN mice. Serum, small intestinal lavage, and fecal lavage samples were collected from 8-9 week-old K/BxN and *Btk^-/y^*/K/BxN mice as in *Methods*. Total IgA was measured by ELISA as in Methods for **(A)** serum, **(B)** small intestinal lavage, and **(C)** fecal lavage. n ≥ 9 mice per group, *p < 0.05, **p < 0.01, Mann-Whitney U test.

Whereas intestinal production of IgA is severely compromised in germ-free mice, serum IgA levels are only reduced by half, suggesting the microbiota contributes to gut IgA production whereas a different process is driving circulating IgA production (6). Serum IgA levels in *Btk*-deficient mice are normal (14). As expected, serum concentrations of IgA were not significantly different between *Btk^-/y^* vs. *Btk^+/y^* K/BxN mice (**Figure 5A**). In contrast, levels of free (non-bacterial-bound) IgA were significantly reduced in small intestinal and fecal lavage harvested from *Btk^-/y^* vs. *Btk^+/y^* K/BxN mice (**Figures 5B, C**). Our data are consistent with separate routes of serum versus intestinal IgA production, and with suboptimal small intestinal villi localization of IgA^+^ plasma cells and their precursors in *Btk*-deficient K/BxN mice.

### Disrupted BTK Signaling Alters CDR3 Polarity, but Not Somatic Hypermutation or Clonal Diversity of Intestinal IgA^+^ Plasma Cells

**Figure 6:**
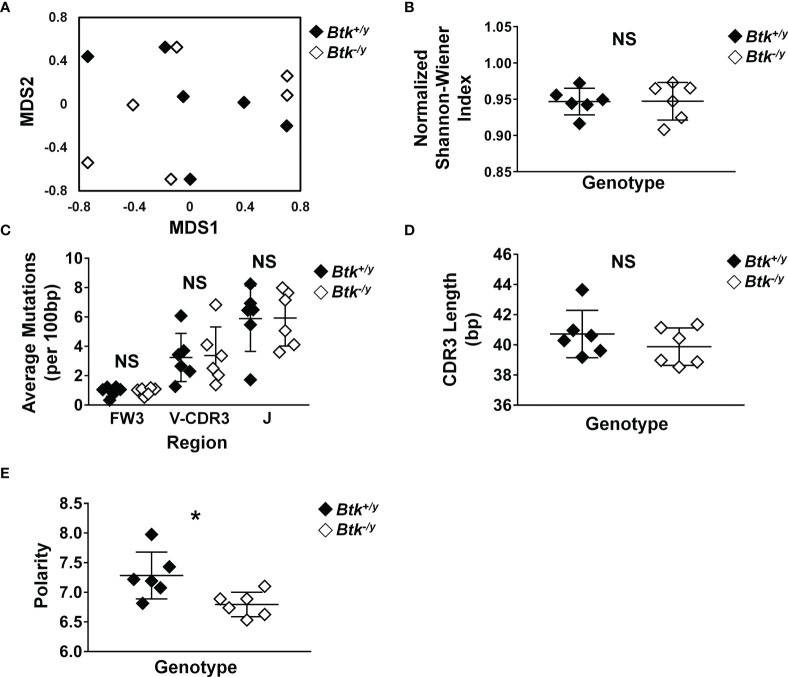
Disrupted BTK signaling does not reduce somatic hypermutation but leads to altered CDR3 polarity in IgA^+^ plasma cells in the small intestinal lamina propria. Lamina propria cells were harvested from the small intestines of K/BxN (black) and *Btk^-/y^*/K/BxN (white) littermates from 3 litters as in Methods, n = 6 mice per group. IgA^+^ plasma cells were identified as live singlet B220^-^ CD19^-^ CD138^+^, surface IgA^-^, intracellular IgA^+^ cells and flow cytometry sorted. Cells were pelleted, DNA was isolated, and IgH sequencing was performed by Adaptive Technologies, as outlined in Methods. An average of 138 productively rearranged sequences were generated per mouse (range: 25-350). Sequence analysis was performed as detailed in Methods. IMGT/HighV-QUEST (http://www.imgt.org/IMGTindex/IMGTHighV-QUEST.php) was used to assign V(D)J genes and assess somatic mutations. VJDtools was used to analyze immunoglobulin gene attributes (https://vdjtools-doc.readthedocs.io/en/master/intro.html). Results were calculated for each individual mouse and are plotted. **(A)** MDS ordination was generated using the Jaccard Index. **(B)** Diversity was calculated using the normalized Shannon-Wiener index. **(C)** Somatic mutations in framework (FW) and complementarity determining regions (CDR) of productively rearranged IgH genes and the average number of mutations per 100 bp was calculated. **(D, E)** VDJtools was used to assess **(D)** CDR3 length and **(E)** amino acid polarity. *p < 0.05, NS, not significant, Mann-Whitney U test.

Intestinal IgA can derive from both T-independent and T-dependent GC responses (5, 6). Given the marked reduction in Peyer’s patch GC B cell numbers (**Figure 1**) and reduction in free IgA present in the small intestinal lumen of *Btk^-/y^* K/BxN (**Figure 5**), we hypothesized the immunoglobulin repertoire of lamina propria IgA plasma cells would be altered. To test this hypothesis, lamina propria cells were isolated from the small intestines of *Btk^+/y^* K/BxN and *Btk^-/y^* K/BxN mice and IgA^+^ plasma cells were flow cytometry-purified as identified in **Figure 3** (B220^-^ CD19^-^ CD138^+^ intracellular IgA^+^ surface IgA^-^). Clear differences between *Btk*-sufficient and *Btk*-deficient IgA plasma cell heavy chain repertoires were not observed by multidimensional scaling (MDS) ordination plotting (**Figure 6A**). Repertoire diversity was unchanged (normalized Shannon-Wiener Index, p = 0.82, Mann-Whitney U test, **Figure 6B**) and there were no differences in the average rates of mutation in either framework region 3 (FW3) or the V or J regions of complementarity determining region 3 (CDR3) (**Figure 6C**) or in amino acid replacement mutations (not shown) between genotypes. A lower rate of mutation was observed in FW3 relative to CDR3, as expected in both groups (p < 0.01 in both genotypes, Mann-Whitney U test). A non-significant trend towards reduced CDR3 length in *Btk* ^-/y^ K/BxN mice was observed (p = 0.48, **Figure 6D**). No difference was observed in CDR3 amino acid charge, disorder, or pH (not shown), but a significant decrease in CDR3 amino acid polarity was observed in IgA^+^ plasma cells isolated from *Btk* ^-/y^ K/BxN mice relative to *Btk^+/y^* mice (average: 6.80 vs. 7.28, p = 0.03, **Figure 6E**). These data indicate that *Btk*-deficient IgA^+^ plasma cells undergo somatic hypermutation but show differences in CDR3 composition, suggesting the small intestinal plasma cell repertoire is qualitatively different when BCR signaling is altered.

### Disruption of Normal B Cell Signaling Decreases IgA Coating of Small Intestinal Commensal Bacteria

**Figure 7:**
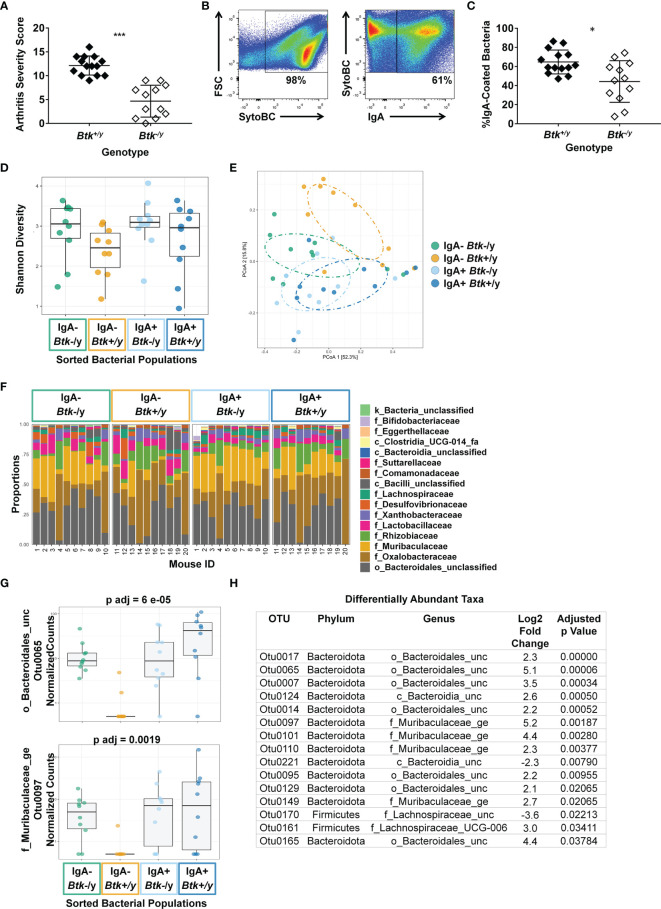
IgA coating of microbes differs between *Btk-*deficient K/BxN mice and *Btk*-sufficient littermates. **(A)** An arthritis severity score was assigned to each paw (0-4) and the total arthritis severity score was calculated (0-16) for n ≥ 12 *Btk^+/y^* (black) or *Btk^-/y^* (white) K/BxN mice per group at the time of small intestinal harvest, ***p < 0.001, Mann-Whitney U test. **(B–H)** Bacteria were harvested from the small intestinal lavage of K/BxN and *Btk^-/y^*/K/BxN littermates from 5 litters that were 8-9 wks old. Cells were stained with SytoBC and anti-IgA antibody and flow cytometry was used to identify and sort IgA-coated or uncoated bacteria. **(B)** Representative plots. **(C)** The frequency of IgA-coated bacteria is plotted for n ≥ 12 individual *Btk^+/y^* and *Btk^-/y^* K/BxN mice per group, *p < 0.05, Mann-Whitney U test. **(D–H)** DNA was isolated from bacteria sorted from *Btk^+/y^* and *Btk^-/y^* K/BxN mice based on being IgA coated or uncoated and microbiome sequencing and analysis was performed by Microbiome Insights using Mothur for the following four groups: *Btk^-/y^* IgA- (green), *Btk^+/y^* IgA- (gold), *Btk^-/y^* IgA+ (light blue), *Btk^+/y^* IgA+ (dark blue). The average number of quality filtered sequence reads per sample was 35446 (Range: 10,000-50,000). **(D)** Shannon diversity for each mouse is plotted; *Btk*: p = 0.06, IgA: p = 0.33, IgA : Btk interaction: p = 0.81, ANOVA. **(E)** Individual mice are plotted based on principle component analysis (PCoA). **(F)** Proportions of the indicated taxa at the family level are plotted for each of n = 10 mice per group. **(G, H)** The DESeq2 package was used to identify differentially abundant taxa among IgA variables using a linear model that included IgA, BTK, and BTK*IgA interactions. Normalized counts for two OTUs and corresponding adjusted p values are shown in **(G)**; other differentially abundant OTUs are shown in **(H)** and (**Figure S1**).

The small intestine is the major site of interface between B lymphocytes and commensal microorganisms in the gut. To test whether BTK signaling loss altered IgA-coating of commensals, bacteria were collected from small intestinal lavage and the frequency of IgA-coated bacteria was assessed as in Methods. Consistent with previous studies (12), *Btk^-/y^* K/BxN mice showed less severe arthritis as compared to *Btk^+/y^* K/BxN mice (average arthritis scores were 4.7 vs. 12.1, respectively, p < 0.001, Mann-Whitney U test, **Figure 7A**). The average proportion of IgA-coated bacteria was reduced in *Btk^-/y^* vs. *Btk^+/y^* K/BxN mice (**Figures 7B, C**, 44% vs. 65%, respectively, p < 0.05). These data show that *Btk* loss impairs IgA-coating of commensal microbes, correlating with disease outcome in this autoimmune arthritis model.

### *Btk*-Deficient Mice Can Produce IgA in Response to Commensal Microbes, but Some Bacteria Escape IgA Coating

IgA-seq (35, 36) was used to assess whether *Btk* loss altered community composition between IgA-coated and uncoated bacteria in the small intestine. No significant differences in Shannon (alpha) diversity were observed, although diversity trended higher in *Btk^-/y^* K/BxN (**Figure 7D**). OTU abundances were summarized into Bray-Curtis dissimilarities and PCoA ordination was performed to obtain graphical representation of microbiome composition similarity among the four groups: IgA^-^/*Btk^-/y^* (green), IgA^-^/*Btk^+/y^* (gold), IgA^+^/*Btk^-/y^* (light blue), IgA^+^/*Btk^+/y^* (dark blue) (**Figure 7E**). Permutational analysis of variance (PERMANOVA) determined significant differences in beta-diversity among IgA (p=0.014), and BTK (p=0.013) factors. IgA-coated and uncoated groups separated by PCoA analysis in *Btk*-sufficient mice and trended toward significance in pairwise analysis (p=0.08); this was expected, as IgA-uncoated bacteria are known to differ from IgA-coated bacteria (35, 36). This contrasts IgA coated and uncoated commensals from *Btk*-deficient mice, which overlap more in the PCoA (p=0.13). IgA-coated bacteria from *Btk*-deficient and -sufficient mice showed near-complete overlap (p=0.13), suggesting *Btk*-deficient B cells can respond to the same microbes that elicit IgA in normal B cells.

**Figure 7F** shows a per sample view of taxonomic composition visualizing the most abundant taxa at the family level. Statistical analysis testing the interaction between *Btk* genotype and IgA coating also identified fifteen differentially-abundant OTUs at the genus level, including *Muribaculaceae* and *Lachnospiraceae* family members, as well as *Bacteroidales* members that were unclassified at the family level (**Figure 7G**). Examples of these differentially abundant OTUs and adjusted p values are shown across all four groups in **Figure 7H**, with all remaining OTUs depicted in **Figure S1**. Twelve of these show a distinct pattern in which the OTU is highly abundant in IgA^+^ fractions from both genotypes, as well as the IgA-negative fraction in the *Btk*-deficient samples, but is not abundant in the IgA-negative fraction of the *Btk*-sufficient samples (**Figures 7G**, **S1**). *Muribaculaceae* are well-represented among these, as are *Bacteroidales*. Overall, these findings indicate that IgA from *Btk*-deficient B cells can bind the same commensal bacteria as IgA from normal B cells, but is deficient, either in abundance or affinity, resulting in inadequate coating, and escape of some microbes that are normally IgA coated into the IgA-uncoated fraction.

## Discussion

The microbiome, shaped by IgA, affects autoimmunity in complex ways (7, 21, 35, 37–40). However, until now the contributions of B lymphocyte signaling to these processes had not been studied. The work presented here uses the *Btk*-deficient K/BxN mouse model to reveal that mucosal GC B cells and intestinal IgA rely in part on this B cell signaling protein. In the absence of *Btk*, appropriate IgA plasma cell localization to small intestinal villi, gut IgA, and IgA coating of commensal bacteria is reduced. Even in this suboptimal state, *Btk*-deficient B cells can still produce bacteria-reactive IgA and generate enough IgA^+^ plasma cells to populate the lamina propria. However, this IgA is functionally incomplete, since some microbes that should be IgA-coated are found to be IgA-free, particularly those in the *Muribaculaceae* and *Bacteroidiales* families. Defective IgA responses may allow commensals which are normally cleared by IgA to flourish in *Btk*-deficient mice, or alternatively may limit the persistence of bacteria that rely on normal IgA for signaling activation or for mucous layer adherence (9, 10), which will require future study.

Commensal microbes are known to be required for autoimmune arthritis to occur in the K/BxN model (22). The findings reported here are therefore somewhat counterintuitive, since *Btk*-deficiency is protective, despite the fact that microbes escaping IgA could hypothetically be more available to trigger disease. Potential explanations for this paradox include the following. First, some commensal microbes use IgA for signaling as well as for their mucus layer retention (10), meaning their fitness may be reduced by ineffective IgA coating. Second, it is possible that there are disease-protective microbes among those that escape IgA coating to persist and compete for gut niches in this model. Third, commensal bacteria live as interdependent consortia, thus altered population coating by IgA may have downstream effects on the health, function, or survival of pathogenic microbes. Finally, it is possible that the ability of *Btk*-deficiency to reduce autoreactive B cells simply overrides any microbiome contributions. These complex bacterial and immune interactions, and their disease effects, are the subject of ongoing studies.

IgA^+^ plasma cells in mice normally arise from innate-like B1 cells from the peritoneal cavity as well as standard B2 B cells present in both Peyer’s patches and isolated lymphoid follicles in the lamina propria (5, 6, 30). 25-50% of intestinal IgA produced in mice is B1 B cell-derived and the remaining 50-75% is produced by B cells from the B2 lineage (5, 6). As in non-autoimmune strains (14), we find a near absence of B1 cells in *Btk*-deficient K/BxN mice, severely limiting their contribution to IgA plasma cell generation. Given these innate-like B cells contribute to T-independent antibody production, the change in CDR3 polarity observed, and the reduction in frequency of commensals that are IgA coated, the IgA antibody repertoire is likely altered by *Btk*-deficiency. B2 cells normally form very large, active GCs in Peyer’s patches. The reduced numbers of GC B cells in Peyer’s patches are consistent with previous work demonstrating equally dramatic reduction of GC B cells in joint-draining popliteal lymph nodes of *Btk*-deficient K/BxN mice (12), and imply a general role for BTK in GC development or function. However, while numbers are reduced, frequencies of IgA^+^ and GC B cells in the Peyer’s patches do not differ, suggesting *Btk*-deficient B cells can still class switch to IgA despite impaired BCR signaling. This is also consistent with *Btk*-deficient T-dependent IgG responses that, while blunted at primary challenge, are adequate following subsequent T-dependent boost (14–16, 18, 41). Preserved T-dependent responses to commensals may similarly support the small reservoir of GC B cells in Peyer’s patches of *Btk*-deficient K/BxN mice. These findings suggest that the remaining intestinal IgA in *Btk*-deficient K/BxN mice is driven by the same commensals that induce IgA under normal conditions. Isolated lymphoid follicles are found in the lamina propria which contain B cells that have a B2 phenotype and serve as IgA plasma cell precursors (30). Our lamina propria isolation protocol likely captures isolated lymphoid follicles. The extent of mutation observed in CDR3 (~5 mutations per 100bp) also suggests B2 origin of IgA^+^ plasma cells, rather than B1, which tend to have lower levels of somatic hypermutation (42). Our findings are also in line with previous data showing that somatic hypermutation still occurs in Peyer’s patch GC B cells even when surface BCR expression and signaling is ablated (43). Differences in clonal diversity were not observed, suggesting *Btk*-deficiency does not restrict immunoglobulin repertoire diversity. Of note, CDR3 analysis has limitations, including the absence of CDR1 and CDR2 analysis, which have historically been more commonly used in clonal and somatic hypermutation analyses, as well as lack of heavy and light chain BCR pairings. Repertoire analysis using single cell sequencing is planned to more fully define the characteristics of IgA that emerges when BTK is absent.

Expression of CD138 and downregulation of B220 and CD19 are classically used to define plasma cells. Plasma cells were additionally phenotypically defined in this study as being intracellular Igκ^+^, confirming they are B lineage cells. We however acknowledge the caveat that this phenotypically defined population was not confirmed to functionally secrete antibody. We detect an additional population of cells which lack the canonical B cell markers, B220 and CD19, yet express a BCR intracellularly based on Igκ positivity. Additional detection of intracellular IgA in the majority of these cells further supports a B lineage origin of these cells. This population of putative CD138^-^ plasma cells shows a modest but not statistically-significant reduction in *Btk*-deficient K/BxN mice. Combined intracellular and extracellular staining for CD138, as well as cell viability dye exclusion suggests CD138-negativity is unlikely due to artifacts from the lamina propria processing protocol, but we cannot fully exclude this possibility. Further studies will be required to confirm whether these CD138-negative lamina propria cells are antibody-secreting cells.

Free IgA was reduced in small and large intestines of *Btk*-deficient K/BxN mice, as was the proportion of IgA-coated bacteria. This initially raised the possibility that commensal-driven IgA production was lacking, perhaps due to impaired GC processes, resulting in B cell failure to respond to bacteria that normally elicit IgA. It was therefore surprising that IgA from *Btk*-deficient mice targeted the same bacteria as normal B cells: there was no difference in IgA-coated community composition between *Btk*-deficient and *Btk*-sufficient K/BxN mice. This indicates BTK is not required to support specific commensal-driven production of IgA, at least in K/BxN mice. However, antigen epitope affinity may be lower or IgA levels may be insufficient to fully cover all of the targeted commensals, which, combined with suboptimal localization of plasma cells may allow some to escape IgA coating, as indicated by the shifting of uncoated IgA samples from *Btk*-deficient mice to more closely resemble IgA-coated fractions. This profile included many OTUs present exclusively in IgA^+^ samples from *Btk*-sufficient animals: 12 individual, differentially-expressed OTUs were almost universally IgA coated in *Btk^+/y^* mice, contrasting their equal abundance in IgA^+^ and IgA^-^ fractions from *Btk* ^-/y^ mice. Colitogenic bacteria were previously shown to be coated with higher levels of IgA, relative to non-pathogenic commensals (44). *Muribaculacea* and *Bacteroidales* members shift into the uncoated fraction in the small intestine of *Btk*-deficient K/BxN mice. Future studies are thus warranted to investigate their impact on arthritis development. Of note, Segmented Filamentous Bacteria (SFB), now known as *Candidata savagella*, drives arthritis in this model *via* T_H_17 enhancement (21), but was not differentially expressed in any of our comparative data. It remains unclear whether *Btk* deficiency impacts IgA affinity for commensals or arthritogenic bacteria.

One additional consideration for this model is that BTK also plays a role in myeloid and mast cells. Although it was previously shown that BTK in myeloid cells was not necessary for arthritis in the K/BxN serum transfer model (12), this question has not been tested in the more robust spontaneous K/BxN model. BTK is important for TLR4 responses to LPS in addition to BCR responses, which is not independently analyzed here, but could be an important contributor (14). Finally, the T cell transgene that drives arthritis in K/BxN mice may also alter commensal immune responses, including, but not limited to, changes in T cell help to B cells.

Clinical use of BTK inhibitors is likely to continue to increase given the number of disease applications for which they are either approved or are being tested. Recent studies show BTK impacts autoreactive-prone B cell maturation, but not survival, and that BTK signaling is needed for normal T-independent type II responses in a timed *Btk* deletion model that mimics the scenario of clinical inhibition (45, 46). Our data suggest perturbation of B cell/IgA/commensal interactions is a likely outcome of BTK inhibition. This should be carefully considered with respect to individual patients, as changes that are beneficial in one setting (e.g. arthritis disease prevention by antibiotic treatment in autoimmune K/BxN mice) can be harmful in another (e.g. type 1 diabetes exacerbation by antibiotic treatment in non-obese diabetic mice) (21, 47). Overall, these data show for the first time that BTK-signaling contributes to IgA development and interaction with commensal bacteria and may alter host interactions with the microbiome.

## Data Availability Statement

The datasets presented in this study can be found in online repositories. The names of the repository/repositories and accession number(s) can be found below: https://www.ncbi.nlm.nih.gov/, BioProject PRJNA706065, https://clients.adaptivebiotech.com/pub/bonami-2021-biorxiv.

## Ethics Statement

The animal study was reviewed and approved by Institutional Animal Care and Use Committee of Vanderbilt University, fully accredited by the AAALAC.

## Author Contributions

PK and RB designed the studies. RB, CT, SV, LN, and BB performed experiments. RB, CT, LN, CW, and AR performed data analysis. RB wrote the manuscript, which PK critically reviewed. All authors provided helpful experimental discussion. All authors approved the final version of this manuscript to be published and agree to be accountable for all aspects of the work in ensuring that questions related to the accuracy or integrity of any part of the work are appropriately investigated and resolved.

## Funding

This work was supported by National Institutes of Health Grants R01-DK-084246 (PK), R01 AI155727 (AR), K12-HD-043483 (RB), and T32-AR-059039 (LN) and the Veteran’s Affairs I01-BX-002882 (PK). This work was also supported through the Vanderbilt Medical Center Flow Cytometry Shared Resource (supported by the Vanderbilt Ingram Cancer Center [P30 CA68485] and the Vanderbilt Digestive Disease Research Center [DK058404]).

## Conflict of Interest

The authors declare that the research was conducted in the absence of any commercial or financial relationships that could be construed as a potential conflict of interest.

## Publisher’s Note

All claims expressed in this article are solely those of the authors and do not necessarily represent those of their affiliated organizations, or those of the publisher, the editors and the reviewers. Any product that may be evaluated in this article, or claim that may be made by its manufacturer, is not guaranteed or endorsed by the publisher.

## Abbreviations

AID, activation-induced cytidine deaminase; ACPA, anti-citrullinated protein antibody; BCR, B cell receptor; BTK, Bruton’s tyrosine kinase; GC, germinal center; MDS, multidimensional scaling; OTUs, operational taxonomic units.
