# Supplementary material for: Bruton’s Tyrosine Kinase Supports Gut Mucosal Immunity and Commensal Microbiome Recognition in Autoimmune Arthritis
Source: Front Immunol. 2022 Mar 29;13:748284. doi: 10.3389/fimmu.2022.748284 (PMC9002138; doi:10.3389/fimmu.2022.748284)
Supplement: Supplementary file 1 [file DataSheet_1.docx]

Supplementary Material

# Supplementary Data


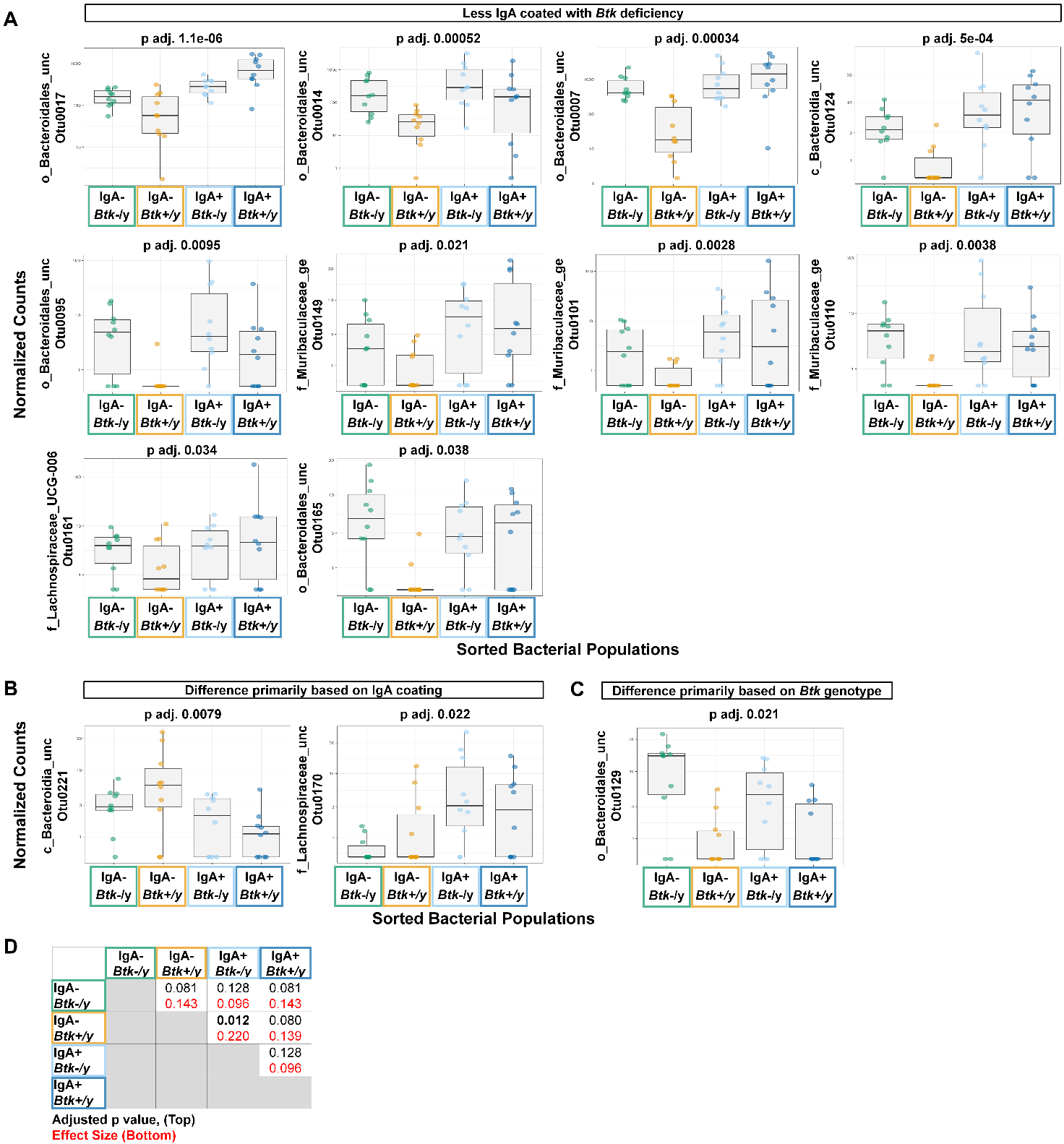


**Supplementary Figure 1. Differentially-abundant bacterial OTUs are identified by IgA-seq analysis of IgA-coated and uncoated commensals from the small intestine of Btk-deficient K/BxN mice and Btk-sufficient littermates.** Bacteria were harvested from the small intestinal lavage of K/BxN and *Btk^-/y^*/K/BxN littermates (n ≥ 12 individual mice per group), prepared as in Methods, and IgA-seq analysis was performed by Microbiome Insights using Mothur as in Fig. 7 for the following four groups: *Btk^-/y^* IgA- (green), *Btk^+/y^* IgA- (gold), *Btk^-/y^* IgA+ (light blue), *Btk^+/y^* IgA+ (dark blue). The DESeq2 package was used to identify differentially abundant taxa among IgA variables using a linear model that included IgA, BTK, and BTK*IgA interactions. Fifteen differentially abundant OTUs were identified; normalized counts for two of these are shown in Fig. 7 and the remaining thirteen OTUs are shown in panels **(A-C)**. Adjusted p values are indicated above each plot, in which individual mice are plotted. These differentially expressed OTUs were grouped as follows: **(A)** Less IgA-coated with *Btk*-deficiency, **(B)** Difference primarily based on IgA coating, and **(C)** Difference primarily based on *Btk* genotype. **(D)** The adjusted p value (black, top) is shown for each pairwise comparison of the four groups using a PERMANOVA (adonis test) along with the effect size (red, bottom).
